# Excision and Regional Flap Reconstruction with Radiation Therapy of Granuloma Annulare of the Digit: A Case Report

**DOI:** 10.1055/a-2665-2132

**Published:** 2025-09-18

**Authors:** Austin Y. Jiang, Linda K. Green, David Netscher

**Affiliations:** 1 Division of Plastic Surgery, Baylor College of Medicine, Houston, Texas; 2 Department of Pathology, Michael E. DeBakey VA Medical Center, Houston, Texas; 3 Department of Pathology, Baylor College of Medicine, Houston, Texas; 4 Department of Orthopedic Surgery, Baylor College of Medicine, Houston, Texas

**Keywords:** granuloma annulare, radiation therapy, finger nodule, radial forearm

## Abstract

Granuloma annulare (GA) is a benign, often self-limited granulomatous skin condition that typically resolves spontaneously. Treatment is often not required, but topical and injectional corticosteroids are the most common interventions. Excision is effective for diagnosis and temporary treatment, but usually not considered curative. This case report describes the unconventional treatment of a recurrent biopsy-proven GA of the index finger. Due to the combination of the recalcitrant nodules interfering with function and patient preference, the nodules were excised multiple times. Ultimately requiring reconstruction with a pedicled radial forearm flap, the patient thereafter received radiation therapy to prevent recurrence, the combination of which has not been previously described in the literature. The patient was treated successfully without evidence of recurrence at 18 months.

## Introduction

Granuloma annulare (GA) is a benign, often self-limited granulomatous skin condition that typically resolves spontaneously. One of the four subtypes of GA is subcutaneous GA, which is characterized by subcutaneous or deep dermal, firm nodules on the scalp and/or extremities. Treatment is often not required, but topical and injectional corticosteroids are the most common interventions. Excision is effective for diagnosis and temporary treatment, but usually not considered curative.

This case report describes the unconventional treatment of a recurrent biopsy-proven GA of the index finger. Due to the combination of the recalcitrant nodules interfering with function and patient preference, the nodules were excised multiple times and ultimately reconstructed with a radial forearm flap, after which the patient received radiation therapy to prevent recurrence.

## Idea

**Fig. 1:**
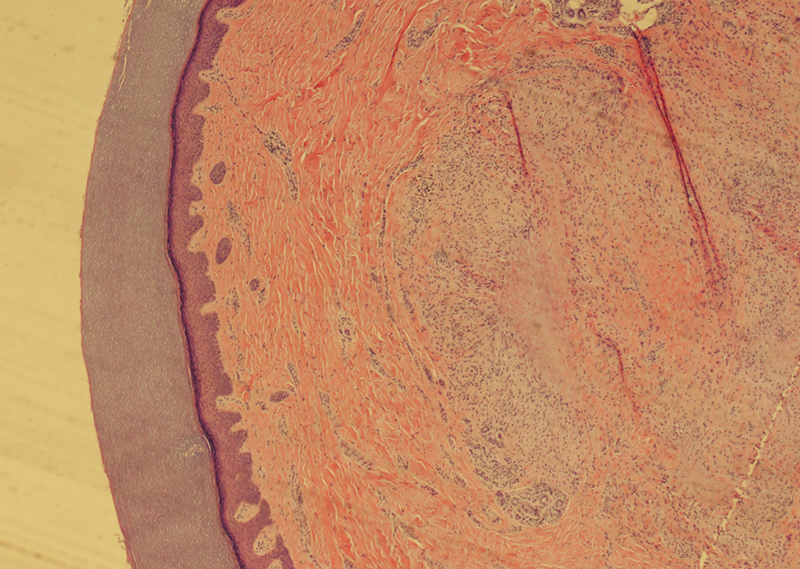
Initial excision of right index finger nodules demonstrates dermal inflammatory infiltrate of necrobiotic degeneration of dermal collagen surrounded by an inflammatory infiltrate of palisading granulomas with scant mucin (1,000 × , H&E stain).

A 48-year-old right-hand-dominant female with a history of type II diabetes mellitus presented to the hand surgery clinic with a 3-month history of an uncomfortable, progressively enlarging 1 cm mass over the dorsum of the proximal interphalangeal (PIP) joint of the right index finger and the right elbow. The PIP mass did affect her function as she had stiffness in this joint, and she was bothered by its cosmesis, and she developed increased tenderness over the previous 6 months as the mass grew in size. This solid lesion had a broad differential, including ringworm or giant cell tumor; thus, an excisional biopsy was performed of both masses. Pathology from both masses returned as necrosis with palisading granulomas ( Fig. 1 ). The differential included GA, an infective process, necrobiosis lipoidica, and dermal rheumatoid nodule. After recovering from this excisional biopsy, the patient developed similar lesions in her elbow and right foot, which were excised with pathologic features consistent with GA.

Two years after excisional biopsy, the patient returned to the clinic with a symptomatic recurrence of three nodules on her right index finger, which persisted at subsequent appointments. Various treatment options were considered, such as intralesional injection with steroids; however, a second excision was chosen for all of the lesions. Laboratory evaluation, including rheumatoid factor, lupus panel (after positive antinuclear antibody testing), sedimentation rate, and uric acid, was undertaken to evaluate for underlying rheumatologic disease and was negative. Excision, which went wide of each lesion, was performed with pathology returning as intermediate to large granulomas with central fibrinous necrosis and palisading histiocytes, differential including GA, rheumatoid nodule, and gouty tophus.

**Fig. 2:**
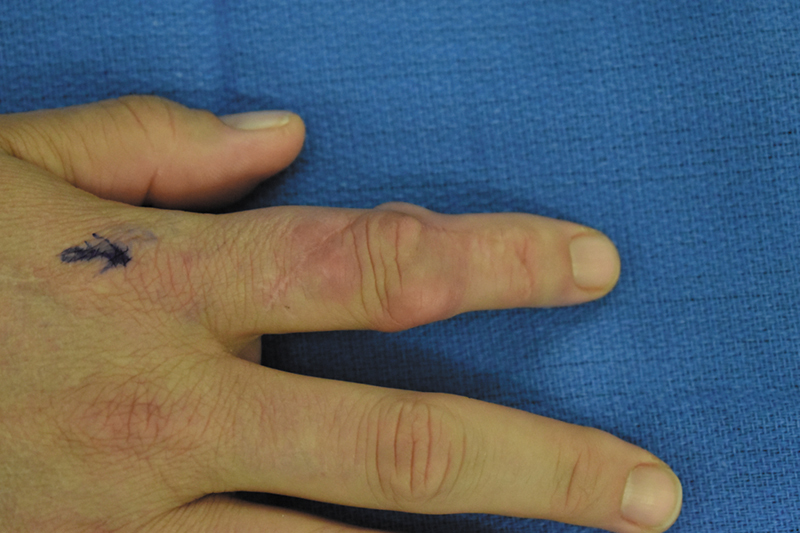
Recurrence of symptomatic right index finger nodule.

A year after the second excision, the patient returned with a new right wrist dorsal non-tender mass and recurrence of subcutaneous confluent nodules at the dorsal PIP, dorsal interphalangeal (DIP), and volar middle phalanx each of which was locally tender to palpation ( Fig. 2 ). The finger masses bothered her most when handling objects such as during cooking and stated it prevented her from completing tasks. Steroid injection in one of the lesions was performed, which did not lead to improvement of pain or a decrease the size. Given the multiple recurrent painful lesions that were affecting tasks of daily living and failure of conservative therapy, the patient desired surgical excision despite being informed of the high risk of further recurrence. Amputation was also discussed, but the patient declined. Rheumatology workup was negative for autoimmune disease, and dermatology agreed with the diagnosis of GA, which had at this point also developed on her feet and buttocks. Given the recurrent nature of this mass and the D.N.'s experience with radiation treatment after surgical management of Dupuytren's contracture, radiation oncology was consulted.

Radiation oncology evaluated the patient and, after a literature review demonstrating limited data on radiation use for GA, they decided to extrapolate from recurrent keloid data to treat the patient with radiation, which the patient desired. Radiation was coordinated to begin after surgical excision of the right index finger masses.

**Fig. 3:**
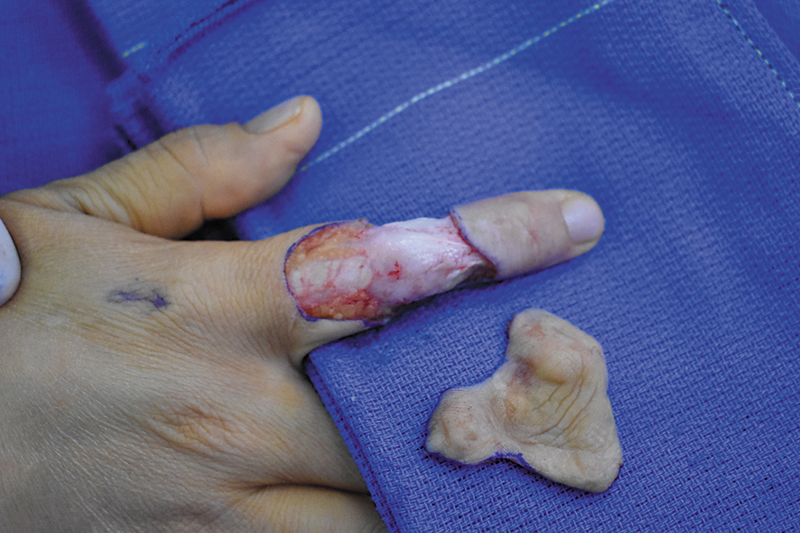
Excision of right index finger nodules.

**Fig. 4:**
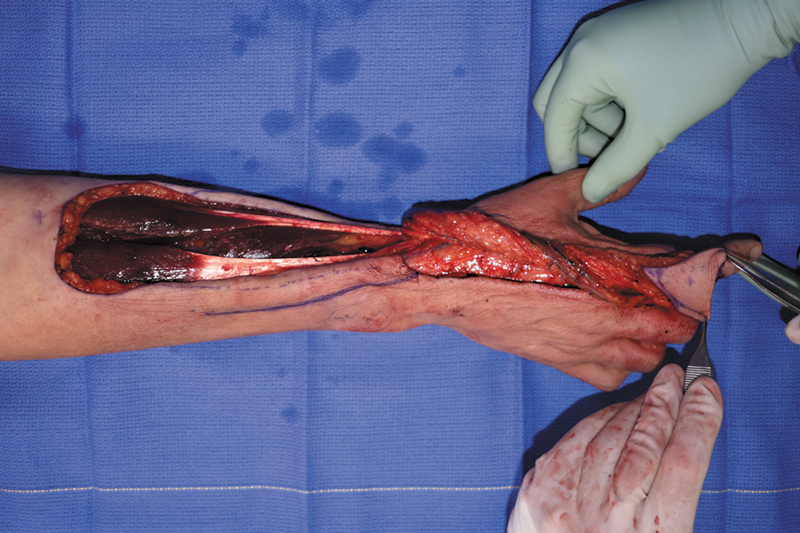
Reconstruction of index finger defect with pedicled radial forearm flap.

**Fig. 5:**
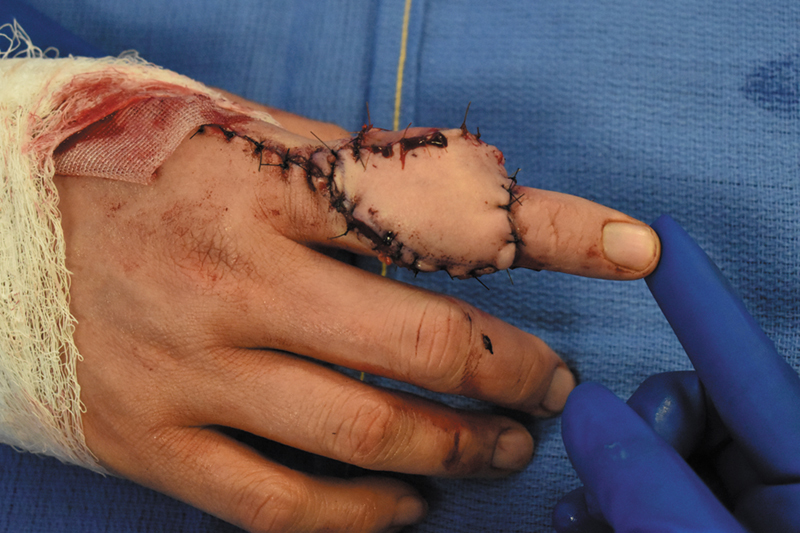
On table result of reconstruction of index finger with pedicled radial forearm flap with skin graft.

Six years after initial presentation, the patient underwent surgical excision of her five right index finger masses and reconstruction of the defect ( Fig. 3 ). Given the size of the defect covering the entire dorsum of the index finger and most of the volar aspect, we opted to perform a pedicled reverse radial forearm island flap to cover the defect ( Figs. 4 and 5 ). Radiation therapy began on postop day 1 with three fractions over 3 days (postop day 1 through 3) for a total of 900 cGy.

**Fig. 6:**
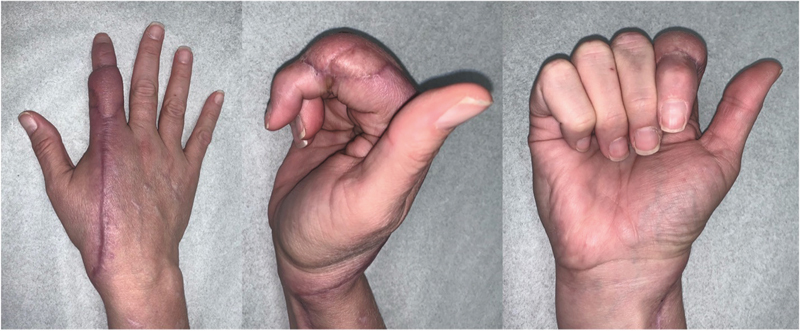
Two-month follow-up photos of reconstruction.

At the time of this publication, the patient was followed up for 35 months postoperatively without recurrence of GA on the right finger ( Fig. 2 ). The patient had swelling of the radial forearm flap postoperatively, which continued to improve along her course and was about 2 cm pulp to palm with a course of physical therapy ( Fig. 6 ). She recovered flexion to about 60 degrees of her metacarpophalangeal joint, 50 degrees of her PIP joint, and 80 degrees of her DIP joint.

Informed consent was obtained from the patient regarding the use of her photos and information.

## Discussion

GA is a benign, often self-limited, granulomatous skin condition characterized by erythematous, annular plaques and papules. The lesions can be found anywhere on the body, and their predilection for particular portions of the body depends on which of the clinical variants it is.

The prevalence of new patients coming to dermatologists with GA is estimated to be 0.1 to 0.4%. 1 Typically presenting in patients between 30 and 50 years of age with a female-to-male ratio of 1 to 2:1, 2 GA is generally asymptomatic but can also present with pruritus and pain. The most common form of GA is localized GA, comprising up to 75% of cases. It presents as a ring of small, firm, flesh-colored or red papules that develop central involution as it progresses. 3 They are most commonly found on the lateral or dorsal surfaces of the hands and feet. Generalized GA is defined as affecting at least the trunk and either upper or lower, or both, extremities. 4 There are flesh-colored papules and plaques that are similar to localized GA. Subcutaneous GA presents as deep dermal or subcutaneous, typically painless, firm nodules on the scalp or extremities, usually in children. 5 6 The finger lesion in this patient had a characteristic clinical appearance of GA most consistent with subcutaneous GA, presenting with only nodules, of which she had multiple in multiple body sites, including the hands, feet, elbow, and buttocks.

Diagnosis of GA is often clinical, but a punch biopsy can be useful if needed. Histopathologic features of GA are dermal or subcutaneous granuloma formation with necrobiosis of collagen, mucin deposition, and histiocytic infiltrate. 1 The etiology of GA is unclear, and many inciting factors have been reported, including trauma, malignancy, viral infections, insect bites, and tuberculosis skin tests. 3 It is believed that it forms due to a type-IV hypersensitivity response to an unidentified antigen. 7 The differential for subcutaneous GA includes rheumatoid nodules, gouty tophus, giant cell tumor, dermoid cyst, pilomatrixoma, lipoma, hemangioma, lymphangioma, osteoma, epithelioid sarcoma, infectious granuloma, and abscesses. 8

Treatment of GA depends on the variant, but most are self-limited and do not require treatment. It is estimated that around 50% of local GA cases will remit spontaneously within 2 years, but recurrence is common. Intralesional injection of triamcinolone is typically used as a first-line therapy, but evidence for its efficacy is limited. Other therapies for GA that also have limited evidence of effectiveness include ultraviolet light phototherapy, cryotherapy, laser therapy, isotretinoin, and TNF-a inhibitors for generalized GA refractory to other therapies. 9 10 11 Surgical excision has been reported as an option for subcutaneous GA, though recurrence is common and is reported to be between 40 and 80%. 2 12 In this case, the patient underwent one steroid injection and two excisions of the lesions on her right index finger, with recurrence and progression of the lesions within 1 year after each excision; therefore, further treatment with unconventional methods was necessary.

Once it was determined that the patient was to undergo excision of all lesions on her index finger, soft tissue coverage was necessary for the resulting defect. The defect extended nearly circumferentially around her finger, sparing only a 1-cm area over her volar middle phalanx, and overlapped the PIP joint. A skin graft of this size in this area would risk contracture. Local coverage options, such as a first dorsal metacarpal artery flap, would not be large enough, making regional or free flaps the best options. This defect required a thin flap that could wrap around the finger. Given the need for a thin, pliable flap capable of wrapping around the finger, donor sites were limited to the dorsalis pedis or groin, both of which would have been equally bulky as the radial forearm. Additionally, using a free flap would have required a similar exposure to access the radial artery; thus, we proceeded with a pedicled radial forearm.

Using radiation therapy for the treatment of GA as an adjuvant after surgical excision has not previously been described in the literature. The recurrent nature of these lesions after surgical excision necessitated an adjuvant therapy. Radiation dosage for treating this case was determined by extrapolating data from keloid treatment. Radiation therapy is suggested to function by inducing antiangiogenic and anti-inflammatory properties, which were deemed suitable for the treatment of an inflammatory condition such as GA. 13 Given the low risk profile for adverse events—such as local malignancy and/or fibrosis or contracture—at this dosage of radiation, the radiation oncology team felt comfortable moving forward.
